# Causes of death in patients with Down syndrome in 2014–2016: A population study in Japan

**DOI:** 10.1002/ajmg.a.62526

**Published:** 2021-10-07

**Authors:** Narumi Motegi, Yui Yamaoka, Akinori Moriichi, Naho Morisaki

**Affiliations:** ^1^ Department of Specific Pediatric Chronic Disease Information National Center for Child Health and Development Tokyo Japan; ^2^ Graduate School of Advanced Integrated Studies in Human Survivability Kyoto University Kyoto Japan; ^3^ Department of Global Health Promotion Tokyo Medical and Dental University Tokyo Japan; ^4^ Department of Social Medicine National Center for Child Health and Development Tokyo Japan

**Keywords:** cause of death, data linkage, Down syndrome, trisomy 21, vital statistics

## Abstract

Despite the higher mortality rates in patients with Down syndrome compared with the general Japanese population, the life span has dramatically increased in Japan and other countries. We aimed to clarify recent causes of death in patients with Down syndrome in Japan. We calculated proportionate mortality and standardized mortality odds ratios (SMORs) among all deaths registered with Down syndrome as the cause of death (ICD‐10 code, Q90) in the Japanese National Death Registry Database in 2014–2016. In the study period, 762 in patients with Down syndrome died. The main causes of death were pneumonia/respiratory infections (20.5%), congenital malformations of the circulatory system (11.2%), other diseases of the circulatory system (9.2%), and aspiration pneumonia (8.4%). The SMORs (95% confidence intervals) were higher for natural death, defined as death of an elderly person with no other cause of death to be mentioned (55.73 [36.92–84.12]), early‐onset Alzheimer's disease, defined as Alzheimer's disease with onset <65 years of age (29.36 [16.44–52.44]), aspiration pneumonia (18.33 [14.03–23.96]), pneumonia/respiratory infections (8.11 [6.76–9.73]), congenital malformations of the circulatory system (8.07 [5.98–10.88]), and leukemia/lymphoma (2.16 [1.55–2.99]) but lower for malignant solid tumors (0.04 [0.02–0.06]) in patients with Down syndrome. Patients with Down syndrome had the greatest relative risk of dying from natural death, early‐onset Alzheimer's disease, and respiratory illnesses, highlighting the need for appropriate medical, health, and welfare services.

## 1 INTRODUCTION

Down syndrome is the most frequent chromosomal abnormality (Weijerman & de Winter, 2010), with a frequency of 12.6/10,000 in the United States (de Graaf et al., 2015) and 28.3/10,000 of all pregnancies in Japan (Sasaki & Sago, 2019; Weijerman & de Winter, 2010). Down syndrome is associated with a variety of congenital malformations, including heart defects (Dennis et al., 2010; Freeman et al., 1998; Irving & Chaudhari, 2012) duodenal atresia, anorectal malformations, hypospadias, and cataracts (Bull & Committee on Genetics, 2011). In addition, Down syndrome is associated with an increased risk of various diseases in each life stage such as neonatal poor feeding, leukemia, and respiratory and otolaryngology infection in childhood and obesity, diabetes mellitus, and stress‐related depressive symptoms in adolescence or adulthood (Bertapelli et al., 2016; Bittles & Glasson, 2004; Melville et al., 2005; Rankin et al., 2012; D. S. Smith, 2001; Stein et al., 2013; Yang et al., 2002).

Studies from Europe, the United States, Sweden, and Denmark show that congenital heart defects, respiratory infections, dementia, pneumonia, and childhood leukemia are main causes of death in patients with Down syndrome, distinct from the leading causes of death observed in the general Japanese population according to recent statistics, which include malignant neoplasms, and heart disease. (Englund et al., 2013; Hasle et al., 2016; O'Leary et al., 2018; Santoro et al., 2021; Yang et al., 2002). However, several studies suggest that the life expectancy of patients with Down syndrome might have increased in many countries including Japan (Motegi et al., 2020; Presson et al., 2013; Yang et al., 2002), in parallel with advances in medical care and welfare. Despite this, the impact of changing demographics of this patient population including racial and ethnic differences (Santoro et al., 2016) on specific medical issues has not been extensively investigated.

We began this study to understand the recent changes in causes of death by age in patients with Down syndrome will allow the identification of the required role of medical, health, and welfare services and to determine the best approaches for treatment. The aim of this study was to clarify the causes of death in patients with Down syndrome in Japan by utilizing Vital Statistics in the 2014–2016 time period.

## 2 MATERIALS AND METHODS

### 2.1 Data source

We extracted data on deaths and stillbirths from the Japanese Vital Statistics Database and on deaths from the online vital statistics submission system database, both maintained by the Ministry of Health and Welfare. The data were extracted on April 26, 2018. The Japanese Vital Statistics System was established in 1899 by the Family Registry Law and Provisions Regarding Notification of Stillbirths and is maintained by the Ministry of Health, Labor, and Welfare. In Japan, all births, deaths, and stillbirths above 12 weeks of gestation must be reported by law. As a general rule, the notification of death should be submitted by the relatives/cohabitants of the deceased to the municipal office with the death certificate from the doctor or the postmortem certificate from the medical examiner or the coroner. The law stipulates such submissions must be submitted within 7 days of the day when the fact of death is known. When the notification of death is submitted to the mayor of a municipality, copies are submitted to the Ministry of Health, Labor, and Welfare. Since 1975, the Statistics and Information Department at the Minister's Secretariat in the Ministry has been responsible for validating the submitted data by cross‐checking with other registries, creating death forms, and choosing International Classification of Disease (ICD) codes for primary cause of death.

The data are then entered into the Vital Statistics Database and used for official statistics. Names, addresses, hospital names, and narrative text about multiple causes of death are not included in this fixed database. In 2007, an additional online vital statistics submission system was established for municipalities, facilitating the direct submission of death certificate data to the Ministry. This database holds all text data on submitted certificates, including name, address, hospital name, and narrative text about multiple causes of death. These two databases are stored separately.

In this study, our analyses included the data on primary causes of death based on ICD‐10 codes for the 2014–2016 period, which were retrieved from the Vital Statistics Database, and the data on contributory causes of death based on narrative text for the 2014–2016 period, which were retrieved from the online vital statistics submission system database.

In our main analysis, we used data from the Vital Statistics Database between January 1, 2014 and December 31, 2016 and identified deaths in which Down syndrome was indicated as the primary cause of death (ICD‐10 code, Q90). These data did not include those in which Down syndrome was registered as a contributory cause of death. Therefore, we complemented our analysis by extracting data from the online vital statistics submission system database for deaths in the 2014–2016 period. We retrieved all data with the word “Trisomy 21” or “Down” in the narrative text on multiple causes of death or contributory cause of death. We then linked these data to the corresponding vital statistics data. The linkage was performed based on the municipal code of the submission location and the certificate number. The distribution of causes of death in the general Japanese population were calculated from the Vital Statistics Database using e‐Stat, an online source for Japanese Governmental Statistic (“e‐Stat of government statistics”).

### 2.2 Classification of causes of death

Two investigators (N.M. and Y.Y.) with medical knowledge categorized the ICD‐coded causes of death among patients with Down syndrome into the following 27 groups: pneumonia/respiratory infections; congenital malformations of the circulatory system; other diseases of the circulatory system; aspiration pneumonia; natural death, defined as the death of an elderly person with no other cause of death to be mentioned; neoplasms; leukemia/lymphoma; early‐onset Alzheimer disease, defined as Alzheimer disease with onset <65 years of age; cerebrovascular diseases; other diseases of the respiratory system; other diseases of the genitourinary system; other infectious diseases; endocrine, nutritional, and metabolic diseases; epilepsy; chronic bronchitis and pulmonary diseases; certain conditions originating in the perinatal period; sepsis; gastrointestinal disease; diseases of liver and biliary tract; other congenital malformations; other diseases including dementia, intellectual disabilities, and nervous system disorders; cerebral palsy, hydrocephalus, and anoxic brain damage; other diseases of the blood and immune system; sudden cardiac death; other respiratory disorders; diseases of the musculoskeletal system and connective tissue; and others. The ICD codes corresponding to these categories are shown in Appendix 1.

### 2.3 Statistical analysis

First, we calculated the number of deaths for specific causes of death and age groups of 10 years (0, 1–9, 10–19, 20–29, 30–39, 40–49, 50–59, 60–69, 70–79, 80–89, and ≥90 years). Second, we selected five disease groups based on the most common causes of death in the present study, namely, aspiration pneumonia, pneumonia/respiratory infection, congenital malformation of the circulation, leukemia/lymphoma, and natural death, as well as two causes of death which previous studies suggest occur in distinctly high and low frequency in patients with Down syndrome, namely early‐onset Alzheimer disease, and malignant solid tumors (Antonarakis et al., 2020; Hasle et al., 2016; Rethore et al., 2020; Yang et al., 2002). We then compared the patients with Down syndrome with the general Japanese population and calculated standardized mortality odds ratios (SMORs) (K. R. Smith & Kliewer, 1995) to assess the age‐specific relationship between causes of death and Down syndrome. SMORs were calculated by dividing the odds for death from a specific cause and the odds for death from all other causes among patients with Down syndrome with the corresponding odds in the general Japanese population (Miettinen & Wang, 1981). Third, we calculated proportionate mortality to determine the probability of death due to a specific cause, calculated as the number of deaths due to a cause divided by the number of living population (Proportionate Mortality, n.d.).

To calculate SMORs, we conducted logistic regression adjusting for age group and year of death (2014–2016). To estimate 95% confidence intervals (CIs) for the SMORs, we assumed that the number of deaths and medical conditions followed a Poisson distribution (Rothman & Greenland, 1998).

The study was approved by the Director‐General of Statistics and the Information Policy Department of the Ministry of Health, Labor, and Welfare (approved on April 26, 2018; approval no. 0426‐3) and the Ethical Review Board of the National Center for Child Health and Development, Tokyo, Japan (approved on October 27, 2016; approval no. 1274).

## 3 RESULTS

### 3.1 Comparison of trends in cause of death between the patients with Down syndrome and the general Japanese population

Between 2014 and 2016, there were 762 and 3,871,196 deaths among the patients with Down syndrome and the general Japanese population, respectively. Table 1 shows the distribution of causes of death among the patients with Down syndrome and in the general Japanese population, based on the major diagnostic categories according to ICD‐10 codes. The most common cause of death in the patients with Down syndrome was diseases of the respiratory system (32.0%), followed by diseases of the circulatory system (13.4%), and congenital malformations, deformations, and chromosomal abnormalities (11.9%). On the other hand, the most common cause of death in the general Japanese population was neoplasms (29.6%), followed by diseases of the circulatory system (26.4%), and diseases of the respiratory system (16.0%).

**TABLE 1:** Causes of death for patients with Down syndrome and general population in Japan in 2014–2016

General Japanese population^a^ (*n* = 3,871,196)			Patients with Down syndrome in Japan (*n* = 762)		
Cause of death	*n*	%	Cause of death	*n*	%
Neoplasms (C00‐D48)	1,145,233	29.6	Diseases of the respiratory system (J00‐J99)	244	32.0
Diseases of the circulatory system (I00‐I99)	1,020,776	26.4	Diseases of the circulatory system (I00‐I99)	102	13.4
Diseases of the respiratory system (J00‐J99)	619,631	16.0	Congenital malformations, deformations, and chromosomal abnormalities (Q00‐Q99)	91	11.9
Symptoms, signs, and abnormal clinical and laboratory findings, not elsewhere classified (R00‐R99)	308,452	8.0	Injury, poisoning, and certain other consequences of external causes (S00‐T98)	50	6.6
Injury, poisoning, and certain other consequences of external causes (S00‐T98)	203,302	5.3	Diseases of the nervous system (G00‐G99)	47	6.2
Diseases of the digestive system (K00‐K93)	144,956	3.7	Neoplasms (C00‐D48)	46	6.0
Diseases of the genitourinary system (N00‐N99)	112,419	2.9	Diseases of the digestive system (K00‐K93)	37	4.9
Diseases of the nervous system (G00‐G99)	92,652	2.4	Symptoms, signs, and abnormal clinical and laboratory findings, not elsewhere classified (R00‐R99)	37	4.9
Certain infectious and parasitic diseases (A00‐B99)	75,908	2.0	Diseases of the genitourinary system (N00‐N99)	27	3.5
Endocrine, nutritional, and metabolic diseases (E00‐E90)	63,339	1.6	Certain infectious and parasitic diseases (A00‐B99)	22	2.9
Mental and behavioral disorders (F00‐F99)	40,055	1.0	Diseases of the blood and blood‐forming organs and certain disorders involving the immune mechanism (D50‐D89)	21	2.8
Diseases of the musculoskeletal system and connective tissue (M00‐M99)	18,537	0.5	Endocrine, nutritional, and metabolic diseases (E00‐E90)	17	2.2
Diseases of the blood and blood‐forming organs and certain disorders involving the immune mechanism (D50‐D89)	13,196	0.3	Certain conditions originating in the perinatal period (P00‐P96)	15	2.0
Congenital malformations, deformations, and chromosomal abnormalities (Q00‐Q99)	6084	0.2	Mental and behavioral disorders (F00‐F99)	3	0.4
Diseases of the skin and subcutaneous tissue (L00‐L99)	4933	0.1	Diseases of the musculoskeletal system and connective tissue (M00‐M99)	3	0.4
Certain conditions originating in the perinatal period (P00‐P96)	1555	0.0	Diseases of the eye and adnexa (H00‐H59) and diseases of the ear and mastoid process (H60‐H95)	0	0.0
Pregnancy, childbirth, and the puerperium (O00‐O99)	113	0.0	Diseases of the skin and subcutaneous tissue (L00‐L99)	0	0.0
Diseases of the eye and adnexa (H00‐H59) and Diseases of the ear and mastoid process (H60‐H95)	55	0.0	Pregnancy, childbirth, and the puerperium (O00‐O99)	0	0.0
Total	3,871,196	100.0	Total	762	100.0

*Note*: Retrieved from https://www.e‐stat.go.jp/classifications/terms/40.

a Numbers provided from e‐Stat of government statistics, n.d.

### 3.2 Cause of death among the patients with Down syndrome

Table 2 shows the causes of death based on disease categories (Appendix 1) and age groups in the patients with Down syndrome. The most common cause of death in the patients with Down syndrome was pneumonia/respiratory infections (20.5%), followed by congenital malformations of the circulatory system (11.2%), other diseases of the circulatory system (9.2%), aspiration pneumonia (8.4%), others (6.7%), and leukemia/lymphoma (5.0%).

**TABLE 2:** Cause of death among patients with Down syndrome in Japan

	Age group (years)																							
	0		1–9		10–19		20–29		30–39		40–49		50–59		60–69		70–79		80–89		≥90		Total	
	*n*	%	*n*	%	*n*	%	*n*	%	*n*	%	*n*	%	*n*	%	*n*	%	*n*	%	*n*	%	*n*	%	*n*	%
Pneumonia/respiratory infections	2	2.0%	4	9.3	0	0.0	2	13.3	4	12.5	20	25.6	38	22.9	75	28.1	11	37.9	0	0.0	0	0.0	156	20.5
Congenital malformations of the circulatory system	26	25.5	13	30.2	6	33.3	4	26.7	9	28.1	15	19.2	8	4.8	4	1.5	0	0.0	0	0.0	0	0.0	85	11.2
Other diseases of the circulatory system	6	5.9	0	0.0	0	0.0	1	6.7	2	6.3	13	16.7	18	10.8	27	10.1	1	3.4	1	16.7	1	16.7	70	9.2
Aspiration pneumonia	1	1.0	1	2.3	0	0.0	0	0.0	0	0.0	3	3.8	22	13.3	33	12.4	4	13.8	0	0.0	0	0.0	64	8.4
Others	7	6.9	3	7.0	3	16.7	1	6.7	0	0.0	2	2.6	17	10.2	17	6.4	1	3.4	0	0.0	0	0.0	51	6.7
Leukemia/lymphoma	21	20.6	4	9.3	4	22.2	2	13.3	2	6.3	2	2.6	2	1.2	1	0.4	0	0.0	0	0.0	0	0.0	38	5.0
Natural death^a^	0	0.0	0	0.0	0	0.0	0	0.0	0	0.0	0	0.0	9	5.4	19	7.1	6	20.7	0	0.0	2	33.3	36	4.7
Cerebrovascular diseases	0	0.0	0	0.0	0	0.0	1	6.7	4	12.5	3	3.8	4	2.4	12	4.5	0	0.0	0	0.0	1	16.7	25	3.3
Neoplasms	0	0.0	2	4.7	1	5.6	1	6.7	1	3.1	5	6.4	7	4.2	3	1.1	1	3.4	2	33.3	1	16.7	24	3.1
Other diseases of the respiratory system	7	6.9	1	2.3	1	5.6	0	0.0	1	3.1	1	1.3	7	4.2	4	1.5	1	3.4	0	0.0	0	0.0	23	3.0
Other diseases of the genitourinary system	2	2.0	0	0.0	0	0.0	0	0.0	1	3.1	3	3.8	3	1.8	9	3.4	1	3.4	1	16.7	0	0.0	20	2.6
Other infectious diseases	1	1.0	2	4.7	0	0.0	0	0.0	1	3.1	1	1.3	1	0.6	12	4.5	0	0.0	0	0.0	0	0.0	18	2.4
Endocrine, nutritional, and metabolic diseases	1	1.0	0	0.0	1	5.6	1	6.7	0	0.0	0	0.0	5	3.0	9	3.4	0	0.0	0	0.0	0	0.0	17	2.2
Epilepsy	0	0.0	0	0.0	1	5.6	1	6.7	0	0.0	3	3.8	5	3.0	7	2.6	0	0.0	0	0.0	0	0.0	17	2.2
Chronic bronchitis and pulmonary diseases	1	1.0	2	4.7	0	0.0	1	6.7	0	0.0	1	1.3	3	1.8	6	2.2	1	3.4	1	16.7	0	0.0	16	2.1
Certain conditions originating in the perinatal period	13	12.7	2	4.7	0	0.0	0	0.0	0	0.0	0	0.0	0	0.0	0	0.0	0	0.0	0	0.0	0	0.0	15	2.0
Sepsis	2	2.0	3	7.0	0	0.0	0	0.0	0	0.0	2	2.6	2	1.2	4	1.5	0	0.0	0	0.0	0	0.0	13	1.7
Early‐onset Alzheimer's disease^b^	0	0.0	0	0.0	0	0.0	0	0.0	0	0.0	0	0.0	4	2.4	8	3.0	0	0.0	0	0.0	0	0.0	12	1.6
Gastrointestinal disease	2	2.0	0	0.0	0	0.0	0	0.0	1	3.1	0	0.0	4	2.4	3	1.1	0	0.0	0	0.0	0	0.0	10	1.3
Diseases of liver and biliary tract	3	2.9	1	2.3	0	0.0	0	0.0	0	0.0	3	3.8	1	0.6	2	0.7	0	0.0	0	0.0	0	0.0	10	1.3
Other congenital malformations	4	3.9	3	7.0	0	0.0	0	0.0	0	0.0	0	0.0	0	0.0	2	0.7	0	0.0	0	0.0	0	0.0	9	1.2
Other diseases including dementia, intellectual disabilities, and nervous system disorders	0	0.0	1	2.3	0	0.0	0	0.0	0	0.0	0	0.0	1	0.6	4	1.5	1	3.4	0	0.0	1	16.7	8	1.0
Cerebral palsy, hydrocephalus, and anoxic brain damage	0	0.0	0	0.0	0	0.0	0	0.0	2	6.3	0	0.0	2	1.2	4	1.5	0	0.0	0	0.0	0	0.0	8	1.0
Other diseases of the blood and immune system	3	2.9	1	2.3	0	0.0	0	0.0	1	3.1	0	0.0	1	0.6	0	0.0	0	0.0	0	0.0	0	0.0	6	0.8
Sudden cardiac death	0	0.0	0	0.0	0	0.0	0	0.0	1	3.1	0	0.0	1	0.6	2	0.7	0	0.0	0	0.0	0	0.0	4	0.5
Other respiratory disorders	0	0.0	0	0.0	1	5.6	0	0.0	1	3.1	0	0.0	1	0.6	0	0.0	1	3.4	0	0.0	0	0.0	4	0.5
Diseases of the musculoskeletal system and connective tissue	0	0.0	0	0.0	0	0.0	0	0.0	1	3.1	1	1.3	0	0.0	0	0.0	0	0.0	1	16.7	0	0.0	3	0.4
Total	102	100	43	100	18	100	15	100	32	100	78	100	166	100	267	100	29	100	6	100	6	100	762	100

^a^ Natural death = Natural death in the elderly, which has no other cause of death to be described.

^b^ Early‐onset Alzheimer's disease = Alzheimer's disease with onset <65 years of age.

The causes of death varied by age. In all age groups under 40 the most common cause of death was congenital malformations of the circulatory system (25.5–33.3%), while in the age groups from 40 to 79 the most common cause of death was pneumonia/respiratory infections (22.9–37.9%). In the 80–89‐year‐age group the most common cause of death was neoplasms (33.3%) and in the over‐90‐year‐age group the most common cause of death was natural death (33.3%).

### 3.3 SMORs and proportionate mortality of specific causes of death in the patients with Down syndrome

We calculated the SMORs for seven disease groups to compare the patients with Down syndrome with the general Japanese population (Table 3). Compared to the general Japanese population, natural death (SMOR: 55.73, 95% CI: 36.92–84.12), early‐onset Alzheimer disease (SMOR: 29.36, 95% CI: 16.44–52.44), aspiration pneumonia (SMOR: 18.33, 95% CI: 14.03–23.96), pneumonia/respiratory infections (SMOR: 8.11, 95% CI: 6.76–9.73), congenital malformations of the circulatory system (SMOR: 8.07, 95% CI: 5.98–10.88), and leukemia/lymphoma (SMOR: 2.16, 95% CI: 1.55–2.99) were significantly more likely to be the main cause of death in the patients with Down syndrome compared to the general Japanese population. On the other hand, the main cause of death was significantly less likely to be malignant solid tumors (SMOR: 0.04, 95% CI: 0.02–0.06) in the patients with Down syndrome than in the general Japanese population.

**TABLE 3:** Standardized mortality odds ratios of select disorders in patients with Down syndrome

Disorder	ICD‐10 code	Observed number of deaths	SMOR	(95% CI)
Natural death^a^	R54	36	55.73	36.92–84.12
Early‐onset Alzheimer's disease^b^	G30.0	12	29.36	16.44–52.44
Aspiration pneumonia	J69.0, K21.0	64	18.33	14.03–23.96
Pneumonia/respiratory infections	J10‐18, J20‐21, J40	156	8.11	6.76–9.73
Congenital malformations of the circulatory system	Q20‐28, I27.0	85	8.07	5.98–10.88
Leukemia/lymphoma	C81‐86, C91‐95, D469, D471	38	2.16	1.55–2.99
Malignant solid tumors	[(C00‐C97) − (C81‐86) + (C91‐C95)]	16	0.04	0.02–0.06

Abbreviations: CI, confidence interval; ICD, International Classification of Disease; SMOR, standardized mortality odd ratio.

^a^ Natural death = Natural death in the elderly, which has no other cause of death to be described.

^b^ Early‐onset Alzheimer's disease = Alzheimer's disease with onset <65 years of age.

**FIGURE 1:**
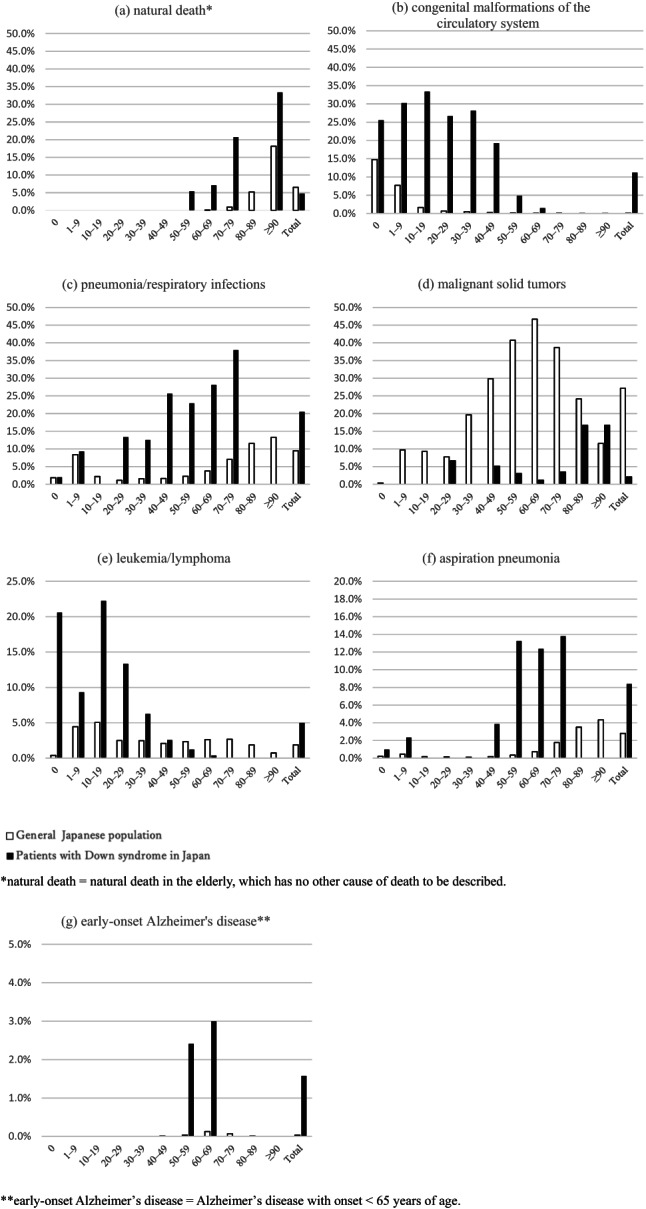
Proportionate mortality for natural death, congenital malformations of the circulatory system, pneumonia/respiratory infections, malignant solid tumors, leukemia/lymphoma, aspiration pneumonia, early‐onset Alzheimer's disease among patients with Down syndrome in Japan (2014–2016)

Figure 1 shows the proportionate mortality for these seven disease categories as causes of death in the patients with Down syndrome and in the general Japanese population. Compared to the general Japanese population, early‐onset Alzheimer disease, aspiration pneumonia, pneumonia/respiratory infections, leukemia/lymphoma, and congenital malformations of the circulatory system were more likely to be a main cause of death whereas natural death and malignant solid tumors were less likely to be a main cause of death in the patients with Down syndrome.

The proportionate mortality for natural death was lower in the patients with Down syndrome (4.7%) compared to the general Japanese population (6.5%). In addition, the patients with Down syndrome who died due to natural death were younger (youngest in the 50–59‐year‐age group) compared to the general Japanese population (youngest in the 60–69‐year‐age group). The proportionate mortality for natural death was consistently higher for all age groups except the 80–89‐year‐age group (50–59 years 5.4% vs. 0.0%, 60–69 years 7.1% vs. 0.1%, 70–79 years 20.7% vs. 0.9%, 80–89 years 0.0% vs. 5.2%, and ≥90 years 33.3% vs. 18.2%).

The proportionate mortality for early‐onset Alzheimer's disease was higher in the patients with Down syndrome (1.6%) than in the general Japanese population (0%) and was concentrated within 50–69 years of age.

The proportionate mortality for aspiration pneumonia and pneumonia/respiratory infections in the patients with Down syndrome (8.4% and 20.5%, respectively) were at least double the proportionate mortality in the general Japanese population (2.8% and 9.5%, respectively). The proportionate mortality was higher for pneumonia/respiratory infections in those between 20 and 79 years of age (0 year 2.0% vs. 1.9%, 1–9 years 9.3% vs. 8.4%, 10–19 years 0.0% vs. 2.2%, 20–29 years 13.3% vs. 1.2%, 30–39 years 12.5% vs. 1.6%, 40–49 years 25.6% vs. 1.6%, 50–59 years 22.9% vs. 2.3%, 60–69 years 28.1% vs. 3.8%, 70–79 years 37.9% vs. 7.0%, 80–89 years 0.0% vs. 11.5%, and ≥90 years 0.0% vs. 13.3%) and consistently higher for aspiration pneumonia for all ages except for the 10–39‐year‐age group (0 year 1.0% vs. 0.2%, 1–9 years 2.3% vs. 0.4%, 10–19 years 0.0% vs. 0.2%, 20–29 years 0.0% vs. 0.1%, 30–39 years 0.0% vs. 0.1%, 40–49 years 3.8% vs. 0.2%, 50–59 years 13.3% vs. 0.3%, 60–69 years 12.4% vs. 0.7%, 70–79 years 13.8% vs. 1.8%, 80–89 years 0.0% vs. 3.5%, and ≥90 years 0.0% vs. 4.3%).

Leukemia/lymphoma was more likely to be the main cause of death in the patients with Down syndrome than in the general Japanese population (5.0% vs. 1.9%) among those aged between 0 and 49 years, with the most notable difference found in the 0‐year‐age group (20.6% vs. 0.4%). Among those over 50 years of age, leukemia/lymphoma was less likely to be the main cause of death in the patients with Down syndrome (1–9 years 9.3% vs. 4.5%, 10–19 years 22.2% vs. 5.0%, 20–29 years 13.3% vs. 2.5%, 30–39 years 6.3% vs. 2.5%, 40–49 years 2.6% vs. 2.1%, 50–59 years 1.2% vs. 2.3%, 60–69 years 0.4% vs. 2.6%, 70–79 years 0.0% vs. 2.7%, 80–89 years 0.0% vs. 1.9%, and ≥90 years 0.0% vs. 0.7%). The proportionate mortality for congenital malformations of the circulatory system was higher in all age ranges except for the over‐70‐year‐age group (0 year 25.5% vs. 14.8%, 1–9 years 30.2% vs. 7.7%, 10–19 years 33.3% vs. 1.6%, 20–29 years 26.7% vs. 0.7%, 30–39 years 28.1% vs. 0.5%, 40–49 years 19.2% vs. 0.3%, 50–59 years 4.8% vs. 0.1%, 60–69 years 1.5% vs. 0.1%, 70–79 years 0.0% vs. 0.1%, 80–89 years 0.0% vs. 0.1%, and ≥90 years 0.0% vs. 0.1%), and overall nearly 100 times higher in the patients with Down syndrome (11.2%) compared to the general Japanese population (0.1%). For malignant solid tumors, the proportionate mortality in the patients with Down syndrome was lower in all age groups except for the over‐90‐year‐age group (0 year 0.0% vs. 0.4%, 1–9 years 0.0% vs. 9.7%, 10–19 years 0.0% vs. 9.3%, 20–29 years 6.7% vs. 7.7%, 30–39 years 0.0% vs. 19.6%, 40–49 years 5.1% vs. 29.8%, 50–59 years 3.0% vs. 40.7%, 60–69 years 1.1% vs. 46.7%, 70–79 years 3.4% vs. 38.7%, 80–89 years 16.7% vs. 24.1%, and ≥90 years 16.7% vs. 11.5%), and overall almost 13 times lower than that in the general Japanese population (2.1–27.1%).

## 4 DISCUSSION

In the present study encompassing the 2014–2016 period in Japan, we found that the main causes of death were diseases of the respiratory and circulatory systems in the patients with Down syndrome. We also observed that mortality due to pneumonia/respiratory infections, aspiration pneumonia, leukemia/lymphoma, early‐onset Alzheimer's disease, and natural death were higher whereas mortality due to malignant solid tumors was lower in the patients with Down syndrome compared to the general Japanese population.

### 4.1 Pneumonia/respiratory infections and aspiration pneumonia

We observed that one in four patients with Down syndrome died from pneumonia and that the proportionate mortality for aspiration pneumonia was higher in the patients with Down syndrome than in the general Japanese population, especially among those aged 50–79 years. Patients with Down syndrome are known to be at a higher risk of infections and are biologically susceptible to developing pneumonia (Ram & Chinen, 2011); therefore, infection prevention, such as vaccination (Beckhaus & Castro‐Rodriguez, 2018), and increased immunity in regular life are important.

Pneumonia has been reported to be the most common cause of death throughout life of patients with Down syndrome in developed countries such as Australia (Bittles et al., 2007) and Sweden (Englund et al., 2013), with a study from the United States reporting an SMOR of 7.61 (95% CI: 7.36–7.87) (Yang et al., 2002) compared with the general Japanese population. This estimate is very close to that estimated in the present study.

Patients with Down syndrome are known to have hypotonicity of orofacial muscles. Thus, early oral care and swallowing training (Hashimoto et al., 2014; Ronald et al., 2011) which have shown to be beneficial to patients with Down syndrome may be useful.

### 4.2 Leukemia/lymphoma

We observed that one in five patients with Down syndrome in the 0‐year‐age group died from leukemia/lymphoma. The SMOR for leukemia/lymphoma was 2.16, consistent with previous studies from the United States (Yang et al., 2002) and Sweden (Englund et al., 2013). The incidence of leukemia is higher in children with Down syndrome under the age of 5 years (Bittles et al., 2007). We observed that deaths due to leukemia occurred more often among those aged 0–29 years, especially among those under the age of 10 years, a finding consistent with the studies from the United States (Yang et al., 2002) and Sweden (Englund et al., 2013).

The high mortality due to leukemia may be explained by the higher incidence rate and the overall lower survival rate observed in children with Down syndrome compared to the general population. Children with Down syndrome are predisposed to acute myeloid leukemia (AML) by 150‐folds (Murphy et al., 2019) and acute lymphoblastic leukemia (ALL) by 20‐folds (Hitzler & Zipursky, 2005) compared to those without Down syndrome. Meanwhile, ALL survival rate is very low (Pennella et al., 2018), and the AML survival rate is slightly high in patients with Down syndrome (Caldwell et al., 2014). In addition, treatment‐related mortality and relapse rate in ALL are higher in children with Down syndrome than in children without Down syndrome (Izraeli et al., 2014).

### 4.3 Early‐onset Alzheimer disease

We observed that death due to early‐onset Alzheimer's disease (onset <65 years of age) was more frequent in patients with Down syndrome compared to the general Japanese population, a finding consistent with the study from the United States (Yang et al., 2002). In our study the SMOR for early‐onset Alzheimer was 29.36, higher from that estimated in a study about 20 years ago (Yang et al., 2002). Another recent study reported that 88% of patients with Down syndrome younger than 65 years of age suffered from dementia (McCarron et al., 2017). These findings suggest that susceptibility to dementia may be has increasing over the years. Given the continuing increase in deaths due to dementia and longer life expectancy in patients with Down syndrome (Englund et al., 2013; Motegi et al., 2020), it is equally probable that the proportionate mortality for Alzheimer's disease associated with advanced age will increase in patients with Down syndrome in future.

While the cause of the observed increased risk for Alzheimer's disease as a cause of death remains unclear, two mechanisms have been proposed. According to the first hypothesis, overproduction of amyloid β due to the overexpression of the amyloid precursor protein (*APP*) gene and overproduction of hydroxyl radicals due to the extra copy of superoxide dismutase 1 *(SOD1*) on chromosome 21 (Perluigi & Butterfield, 2012) underlie the increased risk. Alternatively, obesity, a major risk factor for Alzheimer's disease, is more prevalent among patients with Down syndrome and might contribute to the increased risk (Melville et al., 2005). Prevention of obesity and treatment of Alzheimer's disease from an early stage may lower the risk of dementia in these patients.

The increased SMOR for early‐onset Alzheimer's disease was observed at a higher age range (ages 60–69) compared to the studies from the United States (ages 41–59 (Yang et al., 2002) and Sweden (ages 41–59) (Englund et al., 2013), which might be explained by several factors. First, even if patients with Down syndrome in Japan and other countries develop Alzheimer's disease at a similar age, patients with Down syndrome in Japan may live longer (Motegi et al., 2020). Second, patients with Down syndrome in Japan may develop Alzheimer's disease at a higher age due to genetic or environmental factors. For example, there is a possibility that there are ethnic differences in polymorphisms in *TFAM* (mitochondrial transcription factor A), which encodes a key activator of mitochondrial transcription and is known to be related to the earlier onset of dementia among patients with Down syndrome (Ballard et al., 2016). Third, given that obesity is a known risk for early‐onset Alzheimer's disease (Chuang et al., 2016), cultural differences in eating habits and obesity rates might have contributed to the observed lower disease risk (Ballard et al., 2016; Mazurek & Wyka, 2015).

### 4.4 Natural death

We observed that 4.7% of patients with Down syndrome aged between 50 and 102 years died from natural death. This rate, which was higher than that reported in other countries, was however lower than the percentage of deaths from natural death in the general Japanese population in Japan. In the general Japanese population of Japan, the proportionate mortality of natural death in 2018 was 8.0% (Summary of Vital Statistics, 2018), which is higher than that in Australia (0.2%) and the United States (0.0%). This discrepancy may be due to two factors, long life expectancy of patients with Down syndrome in Japan (Motegi et al., 2020) and ambiguity of the medical definition of natural death. The expected life span of patients with Down syndrome is 50–60 years. In Japan, one in three people die after the age of 60 years; thus, it is possible that more patients with Down syndrome in Japan are dying from natural death compared to that observed in other countries. On the other hand, the rate of natural death is high even in the general Japanese population in Japan, and the high rate of natural death may be a reflection of the culture respecting the dead and the low postmortem examination rates (Maeda et al., 2013). However, there are no previous reports on the susceptibility of natural death in patients with Down syndrome compared to the general population, thus we do not know whether our findings are unique to Japan or the susceptibility has changed over the years.

### 4.5 Malignant solid tumors

Our study showed that only 2.1% of patients with Down syndrome died from malignant solid tumors, which was considerably lower than the rate of almost 30% in the general Japanese population. Similarly, the low frequency of malignant solid tumors in patients with Down syndrome was reported in the United States (Yang et al., 2002). In our study the SMOR for malignant sold tumors was 0.04, close to the estimate in a study about 20 years ago conducted in the United States (Yang et al., 2002) suggesting the susceptibility has not changed over the recent years. Specifically, testicular cancer as a cause of death was lower in patients with Down syndrome in Japan compared to the United States (Yang et al., 2002) and Finland (Patja et al., 2006). The rate of cryptorchidism, an underlying risk for testicular cancers, is known to be high in male patients with Down syndrome and may be fatal if not treated. The low frequency of testicular cancer as a cause of death in patients with Down syndrome may indicate that appropriate surgical measures are followed in Japan.

There are several potential factors that might explain the lower frequency of malignant solid tumors in patients with Down syndrome compared to the general Japanese population. First, patients with Down syndrome are rarely exposed to tobacco, alcohol, and certain occupational exposures that contribute to increased cancer risk. Second, patients with Down syndrome have three copies of tumor suppressor genes on chromosome 21, which contribute to better control of the development of malignant solid tumors (Hattori et al., 2000). Third, cell replication is slower in patients with Down syndrome compared to those without Down syndrome, leading to fewer replication errors in genes involved in tumorigenesis (Yang et al., 2002). Fourth, cells might be more prone to undergo apoptosis in patients with Down syndrome (Nizetic & Groet, 2012). Fifth, patients with Down syndrome generally have a shorter life span and undergo menopause earlier (Bittles & Glasson, 2004). Six, cancer screening rates might be lower in patients with Down syndrome, who are more likely to have intellectual disabilities, which are a known risk for low number and delayed screening (Rethore et al., 2020; Satge et al., 2014). For instance, several studies suggest that patients with Down syndrome might have difficulty in expressing cancer‐related symptoms such as bloody stool, discomfort in diet, fatigue, and body ache due to lower social and communication skills (Mircher et al., 2017; Wiseman et al., 2015).

Provision of seamless transition from pediatric to adult health services is also important. For that purpose, updated guidelines on health care management of adult patients with Down syndrome should be implemented. These guidelines should be understood not only by caregivers including parents but also the patients themselves so that they are aware of any changes in symptoms for early disease detection (Bull & Committee on Genetics, 2011; D. S. Smith, 2001). Future studies should focus on identifying the clinical features and associated social circumstances such as residence, family, and required care at a population‐based level.

This is the first national report on the cause of death in patients with Down syndrome in Japan. The main strength of our study is the utility of national data. However, data obtained from death certificates have several limitations. First, the present study referred to the contents of death certificates and therefore did not include patients with death certificates which did not include Down syndrome as the diagnosis for various reasons. In addition, we cannot deny the possibility that the cause of death might have been misclassified where the true cause of death differed from that stated in the death certificate. Second, we could not differentiate the types of Down syndrome (nondisjunction, translocation, or mosaicism) in the present study, due to the unavailability of relevant data.

## 5 CONCLUSION

Using Vital Statistic Data, we found that the main cause of death were pneumonia/respiratory infections, congenital malformations of the circulatory system, other diseases of the circulatory system, and aspiration pneumonia in the patients with Down syndrome in Japan in the 2014–2016 period. In addition, the patients with Down syndrome were more likely to die from natural death, early‐onset Alzheimer's disease, aspiration pneumonia, pneumonia/respiratory infections, congenital malformations of the circulatory system, and leukemia/lymphoma compared to the general population in Japan. In contrast, the rate of malignant solid tumors as the main cause of death was lower in the patients with Down syndrome compared with the general Japanese population. These results emphasize the need for better medical, and welfare services to meet the needs of patients with Down syndrome of all ages. Further investigation is warranted to understand the associations between the clinical conditions and social factors.

## CONFLICT OF INTEREST

The authors declare that they have no conflicts of interest.

## Supporting information


**Appendix S1** Supplementary information

## Data Availability

Data sharing is not applicable to this article as no new data were created or analyzed in this study.
