# Supplementary material for: Causes of death in patients with Down syndrome in 2014–2016: A population study in Japan
Source: Am J Med Genet A. 2021 Oct 7;188(1):224–36. doi: 10.1002/ajmg.a.62526 (PMC9292866; doi:10.1002/ajmg.a.62526)
Supplement: Supplementary file 1 — Appendix S1 Supplementary information [file AJMG-188-224-s001.docx]

APPENDIX 1. Definition of disorders

| Disorder | ICD-10 code(s) as primary cause of death from the vital statistics database (2014–2016). | Narrative text as contributory cause of death from the online vital statistics submission system database (2014–2016) |
| --- | --- | --- |
| Pneumonia/respiratory infections | J10-18, J20-21, J40 | Pneumonia, pseudomonas pneumoniae |
| Congenital malformations of the circulatory system | Q20-28, I27.0 | Larynx hypoplasia, pulmonary hypertension, endocardial cushion defect, valvular aortic stenosis, complete atrioventricular septal defect, patent ductus arteriosus, primary pulmonary hypertension, interventricular septal defect, atrial septal defect, single ventricle, pulmonary hypertension, congenital cardiac anomaly, persistent pulmonary hypertension of the newborn, Fallot's tetralogy, lymphatic hypoplasia, congenital pulmonary hypertension, left ventricular hypoplasia syndrome, both great vessels from right ventricle, congenital mitral stenosis, atrioventricular septal defect |
| Other diseases of the circulatory system | I21-I25, I269, I31-I51, I713, I809 | Acute circulatory failure, chyle pleural and ascites, malfunction of the heart, congenital chyle chest, abnormal cardiac rhythm, chronic cardiac failure, bilateral chylothorax, chronic congestive heart, failure, lethal arrhythmia, heart failure |
| Aspiration pneumonia | J69.0, K21.0 | Aspiration pneumonia, refractory aspiration pneumonia, dysphagia (cause of death before Down syndrome is aspiration pneumonia or acute pneumonia), deglutition pneumonia, reflux esophagitis |
| Natural death* | R54 | Unable to take oral medications, senility, general debility, dysphagia (cause of death before Down syndrome is senility or not listed), generalized dysfunction, undernutrition |
| Neoplasms | C15-C25, C343, C349, C509, C629, C679, D37-D48, G060 | - |
| Leukemia/lymphoma | C81-86, C91-95, D469, D471 | - |
| Early-onset Alzheimer’s disease** | G30.0 | - |
| Cerebrovascular diseases | I60-69 | Multiple cerebral infarct, aftereffects of cerebral hemorrhage, cerebral hemorrhage |
| Other diseases of the respiratory system | J90-94, J96-98, J852 | Chronic respiratory failure, atelectasis, respiratory failure, acute respiratory failure, lung abscess |
| Other diseases of the genitourinary system | N10-12, N17-19, N039 | Anuria, renal failure, chronic renal failure, acute renal failure |
| Other infectious diseases | A081, A09, B181, B348A, B49, B99, J392, J298, N390 | Neurogenic bladder |
| Endocrine, nutritional, and metabolic diseases | E10-E14, E274, E461, E63-E66, E83-E88 | Malnutrition |
| Epilepsy | G40-G41 | Symptomatic epilepsy, status epilepticus |
| Chronic bronchitis and pulmonary diseases | J42-J47, J84 | Chronic obstructive pulmonary disease, emphysema |
| Certain conditions originating in the perinatal period | P26-28, P59-P60, P78, P92-P96 | Low birth weight infants, fetal edema, septic abortion |
| Sepsis | A403B, A419 | - |
| Gastrointestinal disease | K254, K259, K55-K56 | - |
| Diseases of liver and biliary tract | K72-K75, K802, K810, K922 | Cirrhosis |
| Other congenital malformations | Q32-Q33, Q431, Q820 | Hypoplastic lung |
| Other diseases including dementia, intellectual disabilities, and nervous system disorders | F03, F729, G318, G319, G938 | Dementia |
| Cerebral palsy, hydrocephalus, and anoxic brain damage | G809, G91-G93 | Hydrocephalus |
| Other diseases of the blood and immune system | D693, D70-D76, D849 | Transient abnormal myelopoiesis |
| Sudden cardiac death | I461 | - |
| Other respiratory disorders | G473 | - |
| Diseases of the musculoskeletal system and connective tissue | M311, M512, M600B | - |
| Others | R95, T179, T603, T68-T75, T905, S065A, S131, S397 | Multiple organ failure, disuse syndrome, cachexia, epistaxis, circulatory disorders due to cardiopulmonary arrest, unknown, unlisted |

*Natural death = Natural death in the elderly, which has no other cause of death to be described.

**Early-onset Alzheimer’s disease = Alzheimer’s disease with onset < 65 years of age.
